# Pre-Operative Gastroesophageal Reflux Does Not Affect 30-Day Outcomes in Patients Undergoing Revisional Bariatric Surgery to Single Anastomosis Duodeno-Ileal Bypass (SADI): An Analysis of 933 Metabolic and Bariatric Accreditation and Quality Improvement Program Patients

**DOI:** 10.3390/jcm13206117

**Published:** 2024-10-14

**Authors:** Daniel Meyer, Valentin Mocanu, Noah J. Switzer, Daniel W. Birch, Shahzeer Karmali

**Affiliations:** 1 Centre for Advancement of Surgical Education and Simulation (CASES), Royal Alexandra Hospital, Edmonton, AB T5H 3V9, Canada; 2 Department of Surgery, University of Alberta, Edmonton, AB T6G 2N1, Canada; vmocanu@ualberta.ca

**Keywords:** bariatric, surgery, revision, GERD, SADI, outcomes

## Abstract

**Background:** The use of a single anastomosis duodeno-ileal bypass (SADI) as a revisional procedure in patients with pre-operative GERD is not well understood. Thirty-day outcomes in patients with pre-existing GERD undergoing revision with an SADI have not been previously reported. **Methods:** The Metabolic and Bariatric Accreditation and Quality Improvement Program registry was consulted to identify patients undergoing revisional bariatric surgery with an SADI between 2020 and 2021. Our analysis sought to determine if preoperative GERD had significant impact on thirty-day outcomes. Bivariate and multivariable logistic regression analyses were used to identify independent predictors of 30-day morbidity. **Results:** Preoperative GERD was seen in 342 patients (36.7%). Preoperative GERD was not associated with anastomotic leak (2.5% non-GERD cohort vs. 1.2% GERD cohort; *p* = 0.2) nor bleeding (1% non-GERD cohort vs. 1.8% GERD cohort; *p* = 0.33). There was no difference in thirty-day readmission (5.6% vs. 5.9%, *p* = 0.9), reintervention (2.4% vs. 1.2%, *p* = 0.2), or reoperation (3.6% vs. 2.05%; *p* = 0.19) rates. The multivariable regression analysis revealed that a history of myocardial infarction was associated with a significantly elevated risk of serious complication (OR 12.2; 95% CI 2.79–53.23; *p* = 0.001), as was dyslipidemia (OR 2.2; 95% CI 1.04–4.56; *p* = 0.04). **Conclusions:** Pre-operative GERD does not have any association with anastomotic leak, bleeding, thirty-day readmission, reintervention, or reoperation in patients undergoing revisional bariatric surgery to SADI. A history of myocardial infarction and dyslipidemia are independent predictors of post-operative thirty-day morbidity, irrespective of the presence of preoperative GERD.

## 1. Introduction

Gastroesophageal reflux disease (GERD) is extremely common in patients undergoing revisional [1,2,3] bariatric surgery with rates approaching 50%, particularly in patients who have had sleeve gastrectomy [1,3]. Previous data have demonstrated that preoperative GERD increases both the risks of postoperative complications and readmission in the general surgery [4] and bariatric surgery populations [2,5]. Together, the high prevalence and increased burden of peri-operative complications associated with revisional metabolic surgery make post-bariatric surgery GERD an increasingly common and challenging clinical problem. Despite this challenge, there is currently limited data which can guide practitioners to select the optimal revisional procedure or counsel patients regarding post-operative outcomes.

Introduced in 2007 [6], the single anastomosis duodeno-ileal bypass with sleeve gastrectomy (SADI-S) involves the creation of a gastric sleeve, followed by duodenal mobilization and the establishment of an end-to-side duodenal–jejunal anastomosis [6,7]. This procedure, whether performed in a single- or two-staged manner, has gained popularity due to its efficacy in achieving substantial weight loss and resolving obesity-related medical conditions [8,9] both as a primary and a revisional procedure [10,11,12]. Its safety, metabolic benefit, and anatomic favorability have recently drawn great interest regarding its utilization as a revisional option for post-sleeve related GERD. However, neither the prevalence nor safety of this approach in patients with GERD have yet been characterized.

When considering the prevalence of pre-operative GERD in patients undergoing revisional bariatric surgery, along with the surge in SADI utilization since its endorsement by the American Society of Metabolic and Bariatric Surgeons (ASMBS) in 2020 [13], our study seeks to fill a gap in the literature by investigating whether preoperative GERD influences thirty-day outcomes in patients undergoing revisional bariatric surgery with an SADI to help guide practitioners in selecting patients for SADI, which has not yet been characterized in the literature. Using the Metabolic and Bariatric Accreditation and Quality Improvement Program (MBSAQIP) registry, our aims are to (1) characterize the prevalence of revisional SADI utilization in patients with GERD following primary bariatric surgery and to (2) evaluate the short-term 30-day serious complications and mortality associated with this revisional approach.

## 2. Materials and Methods

### 2.1. Data Source

Data were extracted from the MBSAQIP registry from 2020 to 2021 inclusively. The MBSAQIP is the largest clinical dataset in North American and captures the majority of bariatric procedures across over 900 accredited bariatric centers. Contributing centers are subject to a rigorous independent accreditation process in accordance with internationally recognized bariatric surgical standards and prospective data collection is performed by trained clinical reviewers and encompasses a standardized set of perioperative variables.

### 2.2. Study Design, Variable Definitions, and Population

All patients undergoing revisional bariatric surgery with a single anastomosis duodeno-ileal bypass (SADI) between 2020 and 2021 were included in this retrospective cohort study. Patients were then split into two cohorts, a GERD cohort and non-GERD cohort, based on the presence or absence of pre-operative GERD. The presence of preoperative GERD was determined by the patient’s medical history of established diagnoses and/or dependence on proton pump inhibitors. Our primary outcome was to characterize the population of patients with pre-operative GERD undergoing SADI. Our secondary outcomes were to determine if pre-operative GERD had an influence on thirty-day serious complications after adjusting for comorbidities.

Data collected included patient factors and operative factors. Patient factors included age, sex, body mass index (BMI), functional status (independent, partially dependent, and dependent prior to surgery), smoking status, American Society of Anesthesiologists (ASA) physical status classifications, and comorbidities including diabetes, hypertension, chronic obstructive pulmonary disease, chronic steroid use, renal insufficiency, dialysis, prior venous thromboembolism, therapeutic anticoagulation, oxygen dependence, sleep apnea, prior myocardial infarction, and prior cardiac surgery. We identified the index procedure each patient had undergone leading to their revision, as well as the listed indication for revisional surgery. Technical factors included the operative approach (laparoscopic or open).

Specific postoperative complications included anastomotic leak, bleeding, myocardial infarction, cerebrovascular accident, venous thromboembolism, pulmonary embolism, pneumonia, acute kidney injury, deep and superficial surgical site infections, wound disruption, sepsis, unplanned intubation, and a composite variable of several serious complications. Additionally, 30-day reoperation, intervention, readmission, and mortality rates were assessed. Ethical approval for this retrospective study was not sought due to the use of pre-existing, de-identified data from the MBSAQIP database.

### 2.3. Statistical Analysis

Categorical variables were expressed as absolute values and percentages and univariate analysis was performed using Chi-squared tests. Continuous variables were expressed as weighted means ± standard deviations and the analysis was performed using independent two-sample *t*-tests.

To control for differences between groups and determine the independent influence of pre-operative GERD on 30-day serious complications in revisional SADI patients, a non-parsimonious multivariable logistic regression model was developed using a hypothesis-driven, purposeful selection methodology. Variables from the bivariate analysis with a *p*-value < 0.1, along with those previously identified as clinically relevant to our primary outcome, were included to generate a preliminary main effects model. Models were evaluated using the Brier score and the Receiver Operating Characteristic (ROC) curve. Statistical analysis was performed using STATA 17 statistical software (StataCorp, College Station, TX, USA).

## 3. Results

### 3.1. Basic Demographics and Univariate Analysis

A total of 933 patients in the MBSAQIP data registry underwent revisional bariatric surgery to SADI between 2020 and 2021. Of the 933 patients included, 342 (36.7%) had preoperative GERD (Table 1). Patients with pre-operative GERD were older (46.1 ± 9.6 years vs. 44.5 ± 10.3 years; *p* = 0.03) and had a lower BMI (44.5 ± 8.1 kg/m^2^ vs. 45.6 ± 7.9 kg/m^2^; *p* = 0.049). With regard to metabolic comorbidities, GERD patients had higher rates of treatment-dependent diabetes (20.1% vs. 15.0%; *p* = 0.003), sleep apnea (38.9% vs. 26.9%, *p* < 0.0001), hypertension (45.9% vs. 34.0%; *p* < 0.0001), and dyslipidemia (24.3% vs. 15.7%; *p* = 0.001). There were no significant differences in sex, ASA status, thromboembolic disease, or smoking status between cohorts.

Revision to SADI was most frequently performed for weight recidivism (*n* = 444, 47.6%), followed by inadequate weight loss (*n* = 397, 40.6%), GERD (*n* = 42, 4.5%), and persistent metabolic comorbidities (*n* = 22, 2.3%) (Table 2). The most frequent index procedure was sleeve gastrectomy (*n* = 723, 77.5%) followed by adjustable gastric banding (*n* = 87, 9.3%), and Roux-en-Y gastric bypass (*n* = 82, 8.8%) (Table 3).

### 3.2. Bi-Variate Analysis of Post-Operative Complications

Anastomotic leak was seen in 19 (2%) patients and was not associated with preoperative GERD (2.5% non-GERD cohort vs. 1.2% GERD cohort; *p* = 0.2). Bleeding occurred in twelve (1.3%) patients including six (1%) in the non-GERD cohort and six (1.8%) in the GERD cohort (*p* = 0.33). There was no difference in the rates of AKI, SSI, pneumonia, wound disruption, renal failure, sepsis, pulmonary embolism, myocardial infarction, or overall serious complications (Table 4).

There was no difference in thirty-day readmission (5.6% non-GERD cohort vs. 5.9% GERD cohort; *p* = 0.9), reintervention (2.4% non-GERD cohort vs. 1.2% GERD cohort; *p* = 0.2), or reoperation (3.6% non-GERD cohort vs. 2.05% GERD cohort; *p* = 0.19) rates between the two cohorts.

### 3.3. Multi-Variable Regression Analysis

The multivariable regression analysis revealed that, among all patients undergoing revision to SADI, a history of dyslipidemia was associated with a significantly elevated risk of serious complications (OR 2.2; 95% CI 1.04–4.56; *p* = 0.04), as was a history of myocardial infarction (OR 12.2; 95% CI 2.79–53.23; *p* = 0.001). There were no other significant independent predictors, including GERD, which contributed to serious complications after adjusting for comorbidities (Table 5). The Brier score and receiver operating characteristic of our model were 0.056 and 0.73 respectively, demonstrating good predictive ability of our model.

## 4. Discussion

Using the MBSAQIP database, we present the single largest retrospective study of prospectively collected data examining the impact of pre-operative GERD in patients undergoing revisional bariatric surgery to SADI. We demonstrate that GERD patients were more likely to be older and have a higher burden of metabolic comorbidities than patients without GERD. Nearly half of the revisional SADI patients were revised for weight recidivism and approximately 80% of the index procedures being revised to SADI was sleeve gastrectomy. Lastly, we identify that prior MI history was associated with an over ten-fold increased odds of developing a serious complication following revisional SADI and was the single greatest independent predictor of morbidity.

This study determined that unadjusted thirty-day complications are not significantly different in revisional SADI patients who report preoperative GERD versus those who do not. There was no significant difference in unadjusted complications including anastomotic leak, bleeding, renal failure, myocardial infarction, pneumonia, unplanned intubation, venous thromboembolism, pulmonary embolism, sepsis, or wound infection. There were also no significant differences in the rates of readmission, reintervention, reoperation, nor 30-day mortality. After adjusting for comorbidities, we found no significant association between GERD, or any comorbidity outside of prior myocardial infarction, and serious complications, suggesting that patient factors outside of cardiac risks have a negligible impact on post-operative 30-day morbidity.

Previous data have demonstrated GERD to predispose patients to increased complication risk. Obeid et al. [5] demonstrated that at their institution, Caucasian males undergoing sleeve gastrectomy or Roux-en-Y gastric bypass have a significantly increased length of hospital stay and a readmission rate nearly twice that of patients without GERD, and those admitted had nearly a fivefold higher risk of reoperation. Unfortunately, they did not stratify their data between sleeve gastrectomy and gastric bypass, stating that their study of 533 patients was underpowered for such an analysis. This makes it challenging to generalize our findings to other procedures such as SADI which contain a sleeve gastrectomy as well as a hypoabsortive anastomotic component. Tilak et al. [4] examined the ACS NSQIP database for a variety of abdominal procedures to determine if GERD had any adverse association with outcome in attempt to expand the data from Obeid and colleagues. They identified that patients with GERD were 20.9% more likely to have any complication with the most common being wound and renal complications as well as a tendency toward significance for respiratory and cardiovascular complications [4]. They also identified readmission rates to be higher though reoperation rates did not differ significantly [4]. Our study did not replicate these findings in that we did not demonstrate any difference in readmission or reoperation rate despite a larger sample size. Additionally, we did not demonstrate that the male sex has any impact on thirty-day morbidity suggesting that revisional SADI is safely performed irrespective of GERD, patient sex, and other non-cardiac comorbidities.

The multivariable regression analysis in our study revealed that a history of dyslipidemia and prior myocardial infarction were both significant and independent risk factors for serious complication with odds ratios of 2.2 and 12.2, respectively. The intensity of medical optimization prior to revisional surgery is not characterized in our dataset; however, all MBSAQIP-accredited sites have robust bariatric programs that facilitate medical risk reduction pre-operatively and thus it is likely that these data identify a particularly at-risk population that indeed requires aggressive pre-operative medical optimization. Metabolic and bariatric surgery in patients with non-alcoholic fatty liver disease have been shown to reduce risk of major adverse cardiovascular events after long-term follow-up [14], but it is important to recognize these patients are at an increased perioperative risk; an important finding of our work that has not yet been previously described.

This study has several limitations based on the nature of its design. The first is its retrospective nature along with the fact that data used were from 2020 to 2021 which included cases performed during the COVID-19 pandemic. This may add inclusion or exclusion biases regarding patient selection. Additionally, we were only able to abstract data using the MBSAQIP database’s pre-defined set of variables and are missing important nuanced data such as the method by which preoperative GERD was diagnosed (e.g., clinical diagnosis, PPI dependence, or objective testing with esophagogastroduodenoscopy or ambulatory pH monitoring), how GERD impacted the outcome of their primary procedure, or if preoperative manometry was performed and whether concurrent motility disorder played a role in outcomes from primary or revisional procedures. We additionally do not have any information as to how inadequate weight loss or weight gain was defined. The MBSAQIP database does not provide technical details about the SADI procedure performed including limb lengths and anatomical factors including the presence of a hiatal hernia which may contribute to GERD in our cohorts. The MBSAQIP also does not capture center or surgeon specific practices which may confound our findings. It is also possible that factors outside of what was captured by the database could explain our findings. Lastly, our study cannot comment on clinical change in symptomatic reflux disease following revision to SADI based on our dataset. Previous data have demonstrated that resolution of reflux can be experienced in about 53.2% [15] of patients following SADI, while about 3.6% to 11.9% [15,16] develop de novo reflux and/or reflux esophagitis. The incidence of bile reflux is relatively low at only about 1.23% [17], which is thought to be due to the preservation of the pylorus, similar to that of a traditional sleeve gastrectomy. Further study is required to determine if these patients are at a similar risk for the development of Barrett’s esophagus compared to sleeve gastrectomy patients [18] and whether or not a surveillance esophagogastroduodenoscopy is indicated.

Despite these limitations, our study provides the first characterization of revisional SADI patients with respect to GERD. Taken together, we not only characterize important differences associated with 30-day revisional outcomes and baseline patient factors but identify prior myocardial infarction as the single greatest independent predictor of 30-day serious complications. Further work is needed to identify factors needed to better optimize this at-risk patient group undergoing a revisional SADI.

## 5. Conclusions

Patients with GERD undergoing revisional SADI are older and have higher burden of metabolic comorbidities than those without pre-operative GERD. After adjusting for comorbidities, GERD was not associated with adverse 30-day serious complications; however, prior myocardial infarction was the single greatest independent predictor of morbidity. Patients should be carefully selected for revisional SADI and those with prior cardiac risks should be exhaustively optimized prior to undergoing any revisional procedure with a multidisciplinary approach.

## Figures and Tables

**Table 1:** Basic demographics of patients with and without preoperative reflux prior to revision to SADI.

	No Preoperative Reflux (*n* = 591)	Preoperative Reflux (*n* = 342)	*p*-Value
**Age**			0.069
18–30	34 (5.8%)	8 (2.3%)	
30–40	154 (26.1%)	86 (25.7%)	
40–50	225 (38.1%)	125 (36.6%)	
50–60	126 (21.3%)	92 (26.9%)	
>60	52 (8.8%)	31 (9.1%)	
Range	18–77	20–75	
Mean	44.6 ± 10.32	46.09 ± 9.57	
**Sex**			0.745
Male	92 (15.6%)	56 (16.4%)	
Female	499 (84.4%)	286 (83.6%)	
**BMI**			0.004
<35	28 (4.7%)	28 (8.2%)	
35–40	107 (18.1%)	89 (26.0%)	
40–50	193 (32.7%)	82 (24.0%)	
50–60	125 (21.2%)	69 (20.2%)	
60–70	100 (16.9%)	57 (16.7%)	
>70	38 (6.4%)	17 (5.0%)	
Mean	45.58 ± 7.92	44.51 ± 8.04	
**Functional Status**			
Independent	582 (99.5%)	338 (100%)	
Partially Dependent	3 (0.5%)	0 (0.0%)	
Dependent	0 (0.0%)	0 (0.0%)	
**ASA Category**			0.025
ASA I	0 (0.0%)	1 (0.3%)	
ASA II	129 (21.8%)	48 (14.0%)	
ASA III	433 (73.3%)	278 (81.3%)	
ASA IV	28 (4.7%)	15 (4.4%)	
Not assigned	1 (0.2%)	0 (0.0%)	
**Approach**			
Laparoscopic	586 (99.2%)	338 (98.8%)	0.626
Open	5 (0.9%)	4 (1.2%)	
**Smoking Status**			0.839
Non-Smoker	560 (94.8%)	323 (94.4%)	
Smoker	31 (5.3%)	19 (5.6%)	
**Diabetes**			0.003
Non-diabetic and Diet Controlled	502 (85.0%)	273 (79.8%)	
Non-insulin Dependent	61 (10.3%)	60 (17.5%)	
Insulin Dependent	28 (4.7%)	9 (2.6%)	
**HTN**			<0.000
No HTN	390 (66.0%)	185 (54.1%)	
HTN	201 (34.0%)	157 (45.9%)	
**COPD**			0.024
No COPD	589 (99.7%)	336 (98.3%)	
COPD	2 (0.3%)	6 (1.8%)	
**DLD**			0.001
No DLD	498 (84.3%)	259 (75.7%)	
DLD	93 (15.7%)	83 (24.3%)	
**Chronic Steroids**	11	12	0.118
No chronic steroids	580 (98.1%)	330 (96.5%)	
Chronic steroids	11 (1.9%)	12 (3.5%)	
**Renal Insufficiency**			0.865
No renal insufficiency	587 (99.3%)	340 (99.4%)	
Renal insufficiency	4 (0.7%)	2 (0.6%)	
**Dialysis**			0.187
No dialysis	588 (99.5%)	342 (100.0%)	
Dialysis	3 (0.5%)	0 (0.0%)	
**Prior VTE**			0.025
No prior VTE	569 (96.3%)	318 (93.0%)	
Prior VTE	22 (3.7%)	24 (7.0%)	
**Venous stasis**			0.656
No venous stasis	586 (99.2%)	340 (99.4%)	
Venous stasis	5 (0.8%)	2 (0.6%)	
**Therapeutic Anticoagulation**			0.01
No therapeutic anticoagulation	576 (97.5%)	322 (94.2%)	
Therapeutic anticoagulation	15 (2.5%)	20 (5.8%)	
**Sleep Apnea**			<0.000
No sleep apnea	560 (94.8%)	323 (94.4%)	
Sleep apnea	31 (5.3%)	19 (5.6%)	
**Prior MI**			0.661
No prior MI	584 (98.8%)	339 (99.1%)	
Prior MI	7 (1.2%)	3 (0.9%)	
**Prior Cardiac Surgery**			0.379
No prior cardiac surgery	586 (99.2%)	337 (98.5%)	
Prior cardiac surgery	5 (0.9%)	5 (1.5%)	

BMI—body mass index; ASA—American Society of Anesthesiologists; HTN—hypertension; COPD—chronic obstructive pulmonary disease; DLD—dyslipidemia; VTE—venous thromboembolism; MI—myocardial infarction.

**Table 2:** Primary indication for revisional procedure to SADI.

Indication for Revision	Number (*n*)	Percent (%)
Weight gain	444	47.59
Inadequate weight loss	379	40.62
Gastroesophageal reflux disease	42	4.5
Other	26	2.79
Persistent comorbidities	22	2.36
Adhesions	4	0.43
Mechanical malfunction	4	0.43
Nausea and/or vomiting	3	0.32
Patient intolerance	3	0.32
Dysphagia	2	0.21
Abdominal pain	1	0.11
Fluid, electrolyte, or nutritional depletion	1	0.11
Gastrointestinal tract stricture or obstruction	1	0.11
Patient non-compliance	1	0.11

SADI—Single anastomosis duodeno-ileosotmy.

**Table 3:** Index procedure performed prior to revision to SADI.

Index Procedure	Number (*n*)	Percent (%)
Sleeve gastrectomy	723	77.49
Adjustable gastric band	87	9.32
Roux-en-Y gastric bypass	82	8.79
Adjustable gastric band and sleeve gastrectomy	17	1.82
Gastric stapling (other than VBG)	6	0.64
Sleeve gastrectomy and Roux-en-Y gastric bypass	3	0.32
Adjustable gastric banding and Roux-en-Y gastric bypass	3	0.32
Adjustable gastric banding and other	2	0.21
BPD-DS	2	0.21
Single anastomosis gastric bypass	2	0.21
Other	6	0.64

SADI—Single anastomosis duodeno-ileosotmy; VBG—vertical banded gastroplasty; BPD-DS—Biliopancreatic diversion with duodenal switch.

**Table 4:** Post-operative complications of patients with and without preoperative reflux prior to revision to SADI.

Complication	No Preoperative Reflux (*n* = 591)	Preoperative Reflux (*n* = 342)	*p*-Value
Anastomotic leak	15 (2.5%)	4 (1.2%)	0.154
Bleed	6 (1.0%)	6 (1.8%)	0.334
Reoperation	21 (3.6%)	7 (2.1%)	0.194
Intervention	14 (2.4%)	4 (1.2%)	0.199
Readmission	33 (5.6%)	20 (5.9%)	0.867
Pneumonia	3 (0.5%)	2 (0.6%)	0.876
AKI	0 (0.0%)	0 (0.0%)	-
Deep SSI	15 (2.5%)	7 (2.1%)	0.634
Wound disruption	0 (0.0%)	0 (0.0%)	-
Sepsis	1 (0.2%)	3 (0.6%)	0.28
Unplanned Intubation	2 (0.3%)	0 (0.0%)	0.281
CVA	0 (0.0%)	1 (0.3%)	0.188
VTE	4 (0.7%)	2 (0.6%)	0.865
Serious comp	36 (6.1%)	23 (6.7%)	0.702
Superficial SSI	4 (0.68%)	2 (0.6%)	0.865
Post op deep incisional SSI	4 (0.00%)	3 (0.9%)	0.023
Pulmonary Embolism	0 (0.0%)	1 (0.3%)	0.188
Acute renal failure	0 (0.0%)	0 (0.0%)	-
Myocardial Infarction	0 (0.0%)	0 (0.0%)	-
Post op sepsis	1 (0.2%)	2 (0.6%)	0.28
GI Bleed	3 (0.5%)	0 (0.0%)	0.187
Bowel Obstruction	5 (0.9%)	1 (0.3%)	0.308

AKI—acute kidney injury; SSI—surgical site infection; CVA—cerebrovascular accident; VTE—venous thromboembolism; GI—gastrointestinal.

**Table 5:** Predictors of serious complications in patients with and without preoperative reflux undergoing revision to SADI.

Predictors of Serious Complications	Odds Ratio	95% Confidence Interval	*p*-Value
**Patient Factors**			
Older age (per 10 years)	1.29	0.99–1.66	0.055
Higher BMI (per 5 kg/m^2^)	0.92	0.76–1.12	0.408
Female			
**Comorbidities**			
Diabetes			
Non-Insulin Dependent vs. Non-Diabetic/Diet Controlled	0.96	0.41–2.24	0.929
Insulin Dependent vs. Non-Diabetic/Diet Controlled	0.19	0.22–1.73	0.141
GERD	1.03	0.58–1.84	0.921
DLD	2.17	1.03–4.56	0.04
History of MI	12.2	2.79–53.29	0.001
Therapeutic Anticoagulation	1.39	0.37–5.26	0.624
OSA	0.84	0.43–1.64	0.612
Smoker	0.57	0.13–2.55	0.145
HTN	0.61	0.21–1.2	0.466
**Surgical Factors**			
Operative Length	1.01	1.004–1.01	0.001

BMI—body mass index; GERD—gastroesophageal reflux; DLD—dyslipidemia; MI—myocardial infarction; OSA—obstructive sleep apnea; HTN—hypertension.

## Data Availability

The original contributions presented in the study are included in the article, further inquiries can be directed to the corresponding author.
